# Does monitoring need for care in patients diagnosed with severe mental illness impact on Psychiatric Service Use? Comparison of monitored patients with matched controls

**DOI:** 10.1186/1471-244X-11-45

**Published:** 2011-03-21

**Authors:** Marjan Drukker, Jim van Os, Miriam Dietvorst, Sjoerd Sytema, Ger Driessen, Philippe Delespaul

**Affiliations:** 1 Department of Psychiatry and Psychology, School for Mental Health and NeuroScience MHeNS, Maastricht University, The Netherlands; 2 King's College London, King's Health Partners, Department of Psychosis Studies, Institute of Psychiatry, London, UK; 3 Department of Psychiatry, University Medical Centre Groningen, University of Groningen, Groningen, The Netherlands; 4 Integrated Care Division, Mondriaan, South-Limburg, The Netherlands

## Abstract

**Background:**

Effectiveness of services for patients diagnosed with severe mental illness (SMI) may improve when treatment plans are needs based. A regional Cumulative Needs for Care Monitor (CNCM) introduced diagnostic and evaluative tools, allowing clinicians to explicitly assess patients' needs and negotiate treatment with the patient. We hypothesized that this would change care consumption patterns.

**Methods:**

Psychiatric Case Registers (PCR) register all in-patient and out-patient care in the region. We matched patients in the South-Limburg PCR, where CNCM was in place, with patients from the PCR in the North of the Netherlands (NN), where no CNCM was available. Matching was accomplished using propensity scoring including, amongst others, total care consumption and out-patient care consumption. Date of the CNCM assessment was copied to the matched controls as a hypothetical index date had the CNCM been in place in NN. The difference in care consumption after and before this date (after minus before) was analysed.

**Results:**

Compared with the control region, out-patient care consumption in the CNCM region was significantly higher after the CNCM index date regardless of treatment status at baseline (new, new episode, persistent), whereas a decrease in in-patient care consumption could not be shown.

**Conclusions:**

Monitoring patients may result in different patterns of care by flexibly adjusting level of out-patient care in response to early signs of clinical deterioration.

## Background

There is evidence that the use of person-based rehabilitation strategies improves outcomes in patients diagnosed with severe mental illness (SMI) [1-4]. Such improvements in turn may result in differences in psychiatric service consumption.

SMI is best characterized as a complex combination of psychiatric, somatic, and social needs. Approximately 75% of SMI patients are diagnosed with schizophrenia, psychosis or bipolar disorder [5]. Patients require tailor-made rehabilitation strategies in order to bring about an enduring impact on outcome. However, there is evidence that providers do not always systematically focus on patients' needs but rather select patients for available services [6]. There may be a potential to improve services by introducing need-based treatment plans [7]. This is only possible when needs are routinely and systematically assessed. Therefore, a Cumulative Needs for Care Monitor (CNCM) was introduced in a geographically circumscribed region in the South of the Netherlands to make mental health systems more responsive to individual treatment needs [5]. The CNCM represents a set of diagnostic and evaluative tools that allow clinicians to explicitly evaluate patients' needs and negotiate treatment with the patient [5].

Several recent papers evaluated the use of the CNCM and other related needs assessments in treatment. First, it was shown that identification of unmet needs in the areas of finances, housing and independence with regard to self-care and household skills are followed by targeted action on the part of professional carers [8]. However, need for care in the areas of occupation/daytime activities, psychotic symptoms, psychological distress and self-harm proved more difficult to change from "unmet" to "met" need [8]. Needs are changeable and not only the area of functioning, but also the area of needs requires assessment when evaluating mental health interventions [9]. It has been suggested that systematic needs assessment may produce changes in service outcomes, however prospective research is required [10]. Recent RCTs suggested that systematic needs assessment results in changes in treatment and increased patient satisfaction [2,4], while another study showed associations between needs assessment and patient satisfaction but not with any other outcome [11]. Finally, a multicenter study showed associations between the use of DIALOG, a tool to stimulate patient-carer discussion on 11 domains of need, and improvement in quality of life and unmet needs for care after 12 months [3].

Furthermore, patients at different stages of illness may respond differently to treatment [8]. Patients new in care have acute severe psychopathology, but a relatively intact social network, with higher likelihood of return to pre-onset employment. These first episode patients, particularly those with psychotic disorders, often have low insight and therefore are less likely to formulate specific care needs. Patients in persistent care, however, are more likely to formulate care needs as a result of lack of treatment response and chronic social complications. Therefore, the use of needs-based treatment plans may be associated with different changes in service use depending on treatment status at baseline. A third category is patients in a new episode, defined as having had no care for more than a year, but presenting again after a relapse of previous illness. These patients likely will present with care needs representing a mix of those with first-episode and persistent illness.

Ideally, systematic assessment of needs and other clinical parameters as provided in the CNCM will help clinicians to respond early by making changes in out-patient care, thus preventing further deterioration and hospital admission. Therefore, it was hypothesized that CNCM would be associated with changes indicating more out-patient care and less days in hospital. As different patient groups may respond differently to treatment, we expected that results would depend on duration of treatment status at baseline (no care before 2004; new episode after 365 days out of care; or persistently in care).

### Aims of the study

We examined whether previously reported benefits of monitoring systems are accompanied by changes in psychiatric care consumption. In order to be able to demonstrate changes independent of trends over time (e.g. changes in health care or health care policy) we included patients from a control region in which no systematic and cumulative assessment of needs was in place. The date of the CNCM assessment was also assigned to the matched controls as a hypothetical date of assessment. We hypothesized that care consumption would change after that date in the CNCM region but not in the control region. In particular, we expected an increase in outpatient care and a decrease in inpatient care. Treatment status at baseline was hypothesized to be a modifier of changes in care.

## Methods

### The Cumulative Needs for Care Monitor Database

Mental health professionals (nurses, social workers, psychiatrists, psychologists) are trained to administer CNCM forms aimed to provide clinical case information for use in treatment in negotiation with the patient. Thus, the CNCM monitors treatment in the course of routine care. Data are cumulatively stored and include multiple assessments per patient on needs, psychopathology, well being and functioning of all patients in the region, living both inside and outside hospital. The monitor is part of routine outcome monitoring as required by insurers and health authorities in the Netherlands. It has been approved by the board of directors and executives of the participating care providers. It is allowed to use this data for evaluative purposes and managerial decisions as well as for (anonymised) group comparisons for scientific research. Ethical committees in Maastricht, Utrecht and Groningen have confirmed that by law routine outcome data collected for the purpose of management information is not within their remit as long as patients are aware of the purpose (including scientific publications). Patients are asked during the interview to confirm that the data may be used anonymously for the purpose of research. The interviewer reports the answer on the form. The monitor was introduced in 1998 in a sub region and was expanded to the full region in 2004 (population 660,000) [5].

CNCM forms include various validated clinical instruments: the Camberwell Assessment of Need (CAN) [5,12,13], the Brief Psychiatric Rating Scale (BPRS) [14], the Global Assessment of Functioning Scale, divided into its Psychopathology component and its Impairment component [15], a single item on satisfaction with services, and several brief dimensions of quality of life. Quality of life and satisfaction with services are scored by the patient on 7-point Likert scales; the CAN combines the ratings from both patient and interviewer (see below) and all other instruments are scored by the interviewer [5]. Duration of the interview depends on the level of psychopathology and needs of the patient, but is mostly under one hour.

### Psychiatric Case Registers

Psychiatric Case Registers (PCR) register mental health care consumption of all mental health service users in a region. One of the four Dutch PCRs is active in the CNCM-region of South Limburg [5]. CNCM and PCR data can be matched anonymously at the level of individual patients using an encrypted identification code that is provided through a secure internet connection. This procedure ensures that patient material can be linked to the same person (>99% certainty) without being able to trace information back to specific persons.

The PCR registering service consumption in the 3 provinces in the North of The Netherlands (hereafter: NN, population 1.7 million) was used as a control region, as availability of psychiatric care, level of urbanicity and ethnic diversity (low levels of immigration) is similar to South Limburg. Patients from NN were matched with CNCM patients (see below).

Treatment status at the first mental health contact after July 1^st^, 2004 (hereafter: treatment status at baseline) included three categories: subjects were in care at this date; had never been in care (new patients) or were not in care in the 365 days before this date, but had care before that time (new episode).

### Definition of SMI and MMI

SMI patients had a diagnosis of schizophrenia or non-affective psychotic disorder (DSM IV 295, 297 or 298) or affective psychosis (296, 301.13) or borderline disorder (301.13). In addition, other criteria for SMI were applied because registration of diagnosis is not always complete. Thus, a score of 15 or more on the positive symptom scale of the BPRS defined SMI, as did the combination of impaired functioning (one of the two GAF scales <45; clinicians tend to overestimate the GAF - therefore, the traditional cut-off of GAF scores below 40 for SMI was raised to 45) and need for care in at least two of four *a priori *selected domains (accommodation, welfare benefits, alcohol and drugs). SMI is a patient characteristic: if a patient met criteria at one assessment, he or she was included in the SMI group for all assessments [5].

Patients scoring less than 45 on one of the two GAF scales and presenting with a single need in one of the four *a priori *CAN domains are defined as moderate mental illness (MMI) [5].

### Subjects and matching

The matching procedure and all analyses were performed using the statistical program Stata version 11 [16].

CNCM and PCR data of all South Limburg patients were matched to identify which patients had a CNCM assessment between July 1^st ^and December 31^st ^of the year 2004 and what care they used before July 1^st ^2004. These patients were matched with NN-controls, using propensity score nearest neighbour-matching with replacement (using probit regression estimation method). Propensity scores were based on the following continuous variables: number of days between January 1^st ^1999 and July 1^st ^2004 that patients received (in-patient or out-patient) care, number of hospital days between January 1^st ^1999 and July 1^st^, 2004, date of start mental health care episode in 2004 in days since 1-1-1960 and age, as well as the following categorical variables: gender and treatment status at baseline (defined as: no care before 2004; new episode after 365 days out of care; or persistently in care). All CNCM patients were matched with the NN patient with the nearest propensity score as well as those with the two second nearest scores, aiming to make matching groups consisting of one CNCM and 3 NN patients. However, if more NN patients had the same propensity score, all were included in the matching group.

For each matching group, the assessment date of the CNCM patient was copied to the NN patients as a hypothetical index date had the CNCM been in place in NN. In-patient care consumption, out-patient care consumption and day care in the year before and in the year after this date were obtained from the PCR and used to obtain change scores. NN patients that did not use any care at or after the index date were excluded because patients who were not in care could not have been assessed. Before matching, CNCM patients differed significantly from NN patients with respect to most matching variables (table 1). After matching, no differences remained.

**Table 1:** Propensity score matching results

Before matching				
	***NN*** ***n = 11677***	***CNCM*** ***n = 235***		
	**mean**	**mean**	**t**	**p**

age	40.7 sd = 0.11	42.0 sd = 0.77	-1.65 df = 11910	0.10
# days 1999-2004 that patient received (in- or out-patient) care	720 sd = 6.5	1383 sd = 47.3	-14.24 df = 11910	< 0.001
# in-patient days 1999-2004	170 sd = 4.0	681 sd = 50	-17.7 df = 11910	< 0.001
date of start of care episode in days since 1-1-1960	15624 sd = 6.8	14918 sd = 50.2	14.5 df = 11910	< 0.001
	**%**	**%**	**χ^2^**	**p**

men	42	60	27.5 df = 1	< 0.001
treatment status at baseline			83.5 df = 2	< 0.001
new	29	6		
new episode	11	5		
persistent care	60	89		
age			4.41 df = 3	0.22
18-30 years	21	19		
31-40 years	28	26		
41-50 years	30	28		
51-65 years	22	27		

**After matching**				

	***NN*** ***n = 612***	***CNCM*** ***n = 231***		
	**mean**	**mean**	**t**	**p**

age	42.6 sd = 11.2	42.0 sd = 11.7	0.77 df = 841	0.47
# days 1999-2004 that patient received (in- or out-patient) care	1418 sd = 679	1398 sd = 718	0.37 df = 841	0.71
# in-patient days 1999-2004	696 sd = 776	692 sd = 769	0.07 df = 841	0.95
date of start of care episode in days since 1-1-1960	14886 sd = 736	14904 sd = 763	-0.30 df = 841	0.76
	**%**	**%**	**χ^2^**	**p**

men	60	60	0.0003 df = 1	0.99
treatment status at baseline			0.03 df = 2	0.99
new	5	5		
new episode	5	5		
persistent	90	90		
age^1^			1.86 df = 3	0.60
18-30 years	15	19		
31-40 years	28	26		
41-50 years	28	28		
51-65 years	28	27		

^1 ^Age was included in the matching procedure as a continuous variable. Categories of age are provided for descriptive purpose only

### Statistical analysis

Patients (level 1) were clustered in matched groups (level 2). Therefore, data were subjected to multilevel linear regression analysis, which is ideally suited for analysis of this type of data [17].

Changes in care consumption (after minus before) were the dependent variables in the analyses. As a result, the regression coefficients can be interpreted as the difference in change between the two regions. Region (CNCM or NN) and treatment status at baseline (new; new episode; or persistent care) were included in the model as well as the interaction term between region and treatment status at baseline. Previous treatment was recoded into dummies with persistent-severe as the reference category. When any of the interaction dummies was statistically significant, the Stata Lincom procedure was used to calculate regression coefficients of region for all categories of treatment status at baseline.

## Results

In the matching procedure, 212 matching groups were identified. Two CNCM-patients and their controls were excluded because care consumption of the CNCM patients after the index date was not available. Eighty-five NN patients were excluded because they were not in care at the index date. Because of this, two CNCM patients did not have any controls and were excluded from the analysis. Thus, 208 matched groups were included in the analyses, varying from two to twelve patients, of which 1 to 4 were CNCM patients. A total of 231 CNCM and 612 NN patients were in the final dataset. In the CNCM region, 67.7% was diagnosed with severe mental illness, 22.6% with moderate mental illness and 9.7% with common mental disorder. Thus, ninety percent of the CNCM patients met criteria for severe mental illness (SMI) or moderate mental illness (MMI). Of the CNCM patients, 82% were assessed for the first time, 7% for the second time and 11% for the third to the sixth time. Both in CNCM and in NN, 60% of the patients were male; mean ages were 42.0 and 42.6 years, respectively.

Although patients were matched, in-patient care as well as out-patient care was higher and day care was lower in the CNCM region compared to the NN region, both in the year after and in the year before the index date (table 2).

**Table 2:** Care consumption

	**NN (n = 612)**		**CNCM (n = 231)**		
	**mean (sd)**	**range**	**mean (sd)**	**range**	**t test**
			
**Care consumption after**					
In-patient days	57.12 (125.7)	0 - 365	79.65 (139.7)	0 - 365	t = -2.25*
Out-patient contacts	10.52 (17.9)	0 - 209	17.89 (25.94)	0 - 182	t = -4.67***
Day care	41.33 (94.8)	0 - 365	19.5 (70.3)	0 - 365	t = 3.18**
					
**Difference after minus before**					
In-patient days	-0.12 (44.7)	-348 - 350	-5.2 (63.9)	-324 - 344	t = 1.28
Out-patient contacts	-0.51 (12.3)	-53 - 82	3.41 (20.8)	-71 - 169	t = -3.32**
Day care	-5.31 (67.5)	-363 - 349	-2.63 (58.0)	-313 - 249	t = -0.53

* p < 0.05

** p < 0.01

*** p < 0.001

Comparing care in the year before and the year after the index date suggested that the decrease in in-patient days and the increase in out-patient contacts after the index date was stronger in the CNCM region than in NN (table 3). However, the difference in in-patient days was not statistically significant (β = -5.23, p = 0.17, 95% CI: -12.7; 2.2). Differences in out-patient care (before/after index date) showed an interaction between region and treatment status at baseline (χ^2 ^= 7.17, df = 2, p = 0.03), although there was a significant increase in out-patient care for all 3 categories of treatment status at baseline (new in care β = 11.6, p = 0.04; new episode β = 15.5, p = 0.005; persistent β = 2.8, p = 0.02, table 3).

**Table 3:** Care consumption differences in years before and after index date in CNCM and NN regions

	in-patient days (95% CI)	out-patient contacts (95% CI)	day care (95% CI)
CNCM cf NN: total	-5.23 (-12.7-2.2)		1.78 (-8.0-11.6)
Treatment at baseline* CNCM (interactionterm)	χ^2^= 0.78, df = 2, p = 0.68	χ^2^= 7.17, df = 2, p = 0.03	χ^2^= 3.98, df = 2 p = 0.14
CNCM cf NN: new patients n = 42		11.6* (0.77-22.4)	
CNCM cf NN: new episode n = 40		15.5** (4.59-26.4)	
CNCM cf NN: persistent in care n = 761		2.80* (0.45-5.15)	

* p < 0.05

** p < 0.01

*** p < 0.001

## Discussion

### Methodological issues

Baseline care consumption differed between the CNCM and NN regions. To a degree, these may be attributable to local cultural differences that are difficult to assess. However, because care consumption (capacity of beds) and culture are constant or vary randomly over time, it is possible to control for them by assessing differences in care consumption before and after a given index date, provided the period of observation is not too long.

The present paper has some limitations. First, because neither diagnosis nor level of psychopathology were assessed in the control region, service use is the best indicator of illness severity that was available in both regions and therefore was used for the matching procedure. Because care consumption differs between the regions, it is possible that CNCM patients were matched with less severely ill NN controls. However, this cannot constitute an explanation for the finding that out-patient care use increased after the index date in the CNCM region. In addition, in the control patients, the SMI variable (based on diagnosis or severity) was not available. However, after matching on mental health care use, we assume percentages of SMI are similar to the CNCM patients.

Second, all CNCM patients who were assessed in the second half of 2004 (6 months) were included in the matching procedure. Because the CNCM was expanded to the full South Limburg region in the first half of 2004, there were more patients assessed in this time period than in the year 2003 (12 months). Because PCR data were available until the end of 2005, patients assessed in the first half of 2005 could not be followed for a full year and were, therefore, not included in the matching. This resulted in a relatively high proportion of first assessments, but of all these patients, the ones who remained in care had later follow-up assessments. In theory, changes in service provision may occur more often after the first assessment, as previously unknown needs more often may come to light. In addition, a small group of patients with common, less severe mental disorders, outside the range of SMI or MMI, were not excluded to avoid a loss of power and, in addition, because it may be argued that all patients treated in mental health services represent a selection based on severity, given that only the more severe half of psychiatric patients is treated by mental health professionals, rather than the GP [18].

Currently, a CNCM-like assessment is also in place in NN. However, assessments started only in 2007. Thus, results of the present paper are not biased by this new practice.

Finally, two other differences between the CNCM-region and NN may have impacted on the results. First, the CNCM region was expanded in the beginning of 2004. Therefore, during this period, most patients were assessed for the first time. Second, in a sub region of the CNCM, Function Assertive Community Treatment (FACT) was in place since 2002, and FACT is associated with different patterns of psychiatric care consumption [19]. *Post-hoc *sensitivity analyses, in which patients from the FACT region and their controls were excluded, showed results similar to the original analyses. Out-patient care only increased in the new episode patients (β = 13.3, p = 0.01), but not in the new or the persistent patients (β = -1; β = 0.25, for new and persistent patients respectively); there were no significant differences in in-patient care (β = -8.5, p = 0.16) and day care (β = 4.6, p = 0.5).

### Explaining the results

That out-patient care increased in the year after the index date is likely to be a consequence of treatment in the CNCM region. We hypothesized that an increase in out-patient care would prevent admission, by delivering differentiated need-based care rather than standard admission. However, in the present analyses, the increase in out-patient care did not go together with a decrease in in-patient care.

The present results are based on "*real-life*" clinical practice as opposed to randomized controlled trials (RCT), which generally study selected subsamples of patients without comorbidity and addiction problems. Previously, an RCT did not show an association between a needs-assessment and hospital admissions, but this RCT did not involve clinicians in the assessment [11]. Although we also did not find evidence for changes in in-patient care, but only in out-patient care, we feel that involvement of clinicians in the assessment is crucial. This is the core feature in the CNCM, and is hypothesized to contribute to the observed effects as behavioural change of clinicians, as induced by the CNCM, is required to induce changes in care. Two RCTs on two different need-for-care instruments, developed to improve communication between clinicians and patients, both showed that treatment changed more in the intervention group [2,3]. Furthermore, a *real-life *observational study showed that patients who were treated in a self-help program used less in-patient care but more care in total, suggesting an increase in out-patient care [20]. A limitation of this latter study was that subjects themselves choose to participate or not, so that self-help and control group had different characteristics [20], which may explain why the difference in care consumption was not accompanied by improved outcomes [20]. However, a multicenter RCT did provide evidence that changes in treatment were accompanied by improvement in functioning and quality of life [3]. Thus, improved communication through systematic need for care assessment may lead to different patterns of care consumption which may contribute to improved outcomes.

### Capacity of out-patient and in-patient care

The fact that the observed increase in out-patient care was not accompanied by a decrease in in-patient care may be a consequence of the bed capacity in the region. The differences in care consumption between the CNCM and NN regions may indicate an overcapacity of in-patient beds in the CNCM region. It has been shown that the introduction of community treatment in a region impacts less on reduction of hospital days in new patients if the number of beds is not reduced [21]. It has been reported that patients receive treatment because it is available, rather than because of an actual need for care [22]. Professional carers should assign patients to inpatient and outpatient treatment, based on need based treatment plans as described in the present paper. Ideally, this is in the context of team-based community care, with the possibility to deliver services flexibly across in-patient and out-patient care solutions. This way the availability of in-patient or out-patient care is easier to adapt to the needs in the patient population. However, the health care system may not have this flexibility.

## Conclusion

The present paper showed evidence for differences in out-patient care consumption as a result of the use of CNCM assessments and feedback in treatment. Previous papers evaluating the CNCM also showed differences in outcomes [8] and therefore evidence that CNCM and other need assessment systems works positively is accumulating. It may be recommended to introduce CNCM-like monitors in other regions for the evaluation of patients' needs as well as the negotiation of treatment, but more research is needed. An important question is whether the reported improvements are cost-effective.

## List of abbreviations

BPRS: Brief Psychiatric Rating Scale; CAN: Camberwell Assessment of Need; CNCM: Cumulative Needs for Care Monitor; df: degrees of freedom; FACT: Function Assertive Community Treatment; MMI: Moderate mental illness; NN: North of the Netherlands; PCR: Psychiatric Case Registers; RCT: Randomized controlled trials; sd: standard deviation; SMI: Severe mental illness.

## Competing interests

The authors declare that they have no competing interests.

## Authors' contributions

MDr and MDi performed the analyses. MDr wrote the paper; MDi added various paragraphs and edited the paper. JvO and PhD are scientific coordinators of the CNCM and supervised this paper as it uses CNCM data. JvO revised the paper. PhD edited the final draft and wrote various paragraphs. SS and GD were responsible for the PCR data in NN and in the CNCM region, respectively, and they edited the final draft. All authors read and approved the final manuscript.

## Pre-publication history

The pre-publication history for this paper can be accessed here:

http://www.biomedcentral.com/1471-244X/11/45/prepub
